# Risk factors for gallstone disease in Shanghai: An observational study

**DOI:** 10.1097/MD.0000000000018754

**Published:** 2020-01-17

**Authors:** Qiyun Gu, Guoqing Zhou, Tao Xu

**Affiliations:** a Department of General Surgery, Jinshan Branch of the Sixth People's Hospital of Shanghai; b Department of Medical Examination, Jinshan Hospital Affiliated to Fudan University, Shanghai, China.

**Keywords:** gallstones disease, retrospective study, risk factor

## Abstract

The purpose of this study was to evaluate related risk factors for gallstone disease in Shanghai.

We analyzed successive physical examinations of 2288 adults who were recruited at the Jinshan Branch of the Sixth People's Hospital of Shanghai and Jinshan Hospital Affiliated to Fudan University Hospital from July 2010 to December 2012. The odds ratios (ORs) with 95% confidence intervals (CIs) were used to measure the influence factors on the risks of gallstone development.

The incidence of gallstone disease was 4.11% (94/2,288). Older age (OR: 1.02; 95% CI: 1.00–1.03; *P* = .039), higher body weight (OR: 1.02; 95% CI: 1.00–1.04; *P* = .021), alanine transaminase activity (ALT) (OR: 1.02; 95% CI: 1.01–1.03; *P* = .001), total standard bicarbonate (SB) (OR: 1.04; 95% CI: 1.02–1.06; *P* < .001), free SB (OR: 1.17; 95% CI: 1.12–1.21; *P* < .001), and low density lipoprotein (LDL) levels (OR: 1.59; 95% CI: 1.32–1.91; *P* < .001) were associated with an increased risk of gallstone disease. Based on univariate logistic analysis, increased triglyceride (TG) levels were associated with a reduced risk of gallstone disease (OR: 0.76; 95% CI: 0.60–0.97; *P* = .024). The results of multivariable logistic regression analysis showed higher LDL levels correlated with an increased risk of gallstone disease (OR: 1.92; 95% CI: 1.31–2.81; *P* < .001), while age, weight, ALT, total SB, free SB, and TG levels did not affect the risk of gallstone disease.

The although unadjusted results showed age, weight, ALT, total SB, free SB, TG, and LDL levels to be associated with the risk of gallstone disease, adjusting for potential factors revealed only increased LDL levels to be associated with an increased risk of gallstone disease.

## 1 Introduction

Gallstone disease is the most common disorder of the biliary system,^[1]^ which accounts for 10% to 15% prevalence in developed countries,^[2]^ and 3% to 11% in different regions of China.^[3]^ Although the disease is generally non-life-threatening, the quality of life for patients is affected by upper right abdominal pain with an increased incidence of nausea, vomiting, and feelings of fullness after meals.^[4]^ The incidence of gallstone disease has increased rapidly by nearly 2-fold every 10 years due to living conditions, changes in diet, the widespread use of type-B ultrasound, the popularity of health care, and other factors.^[5]^ Therefore, further exploration of potential risk factors and the incidence of gallstone disease should be conducted.

The types of gallstones are mainly divided into cholesterol and pigment stones. Cholesterol stones account for 80% to 90% of gallstones in Europeans and Americans, and cholesterol crystals precipitating from oversaturated bile reflects a high level of cholesterol secretion from the liver.^[6]^ Further, pigment stones are mainly observed in Asians and can be divided into high-residue, low-residue, and mixed pigment stones, which form calcium bilirubinate and polymerized bilirubin, and they are usually related to hemolysis, bacterial infection, or liver disease.^[7–9]^ Although some patients may not have any symptoms, numerous patients experience acute cholecystitis or gallstones may even become lodged in the bile duct. If the factors associated with gallstone disease could be identified, more work could be done to prevent its development and associated complications.^[10,11]^

Systematically evaluating the risk factors for increased incidence of gallstone disease in Shanghai is particularly important due to Shanghai's position as one of the most prosperous regions in China, which provides guidance to other regions. The purpose of this study is based on single-center data from physical examinations in the Jinshan District of Shanghai, and it explores the incidence of gallstone disease and potential risk factors, which could provide evidence for prevention of gallstones.

## 2 Methods

### 2.1 Subjects

Retrospective data was acquired from continuous physical examinations of adults at Jinshan Branch of the Sixth People's Hospital of Shanghai and Jinshan Hospital Affiliated to Fudan University Hospital from July 1, 2010, to December 31, 2012. The study was approved by the Institutional Review Board of Jinshan Branch of the Sixth People's Hospital of Shanghai. A total of 2312 subjects were recruited, and participants with a history of cholecystectomy were excluded (n = 24). Finally, 2288 individuals were included in the study.

### 2.2 Measurement

Patients came to the hospital in the morning on an empty stomach for blood collection. Five milliliters of blood was collected from each patient and treated with an anticoagulant. The blood samples were then measured in accredited laboratories. An automatic biochemical analyzer (ARCHITECT c16000) was used to measure total cholesterol (TC), triglyceride (TG), alanine aminotransferase (ALT), low-density lipoprotein (LDL), high-density lipoprotein (HDL), and fasting blood glucose (FBG) levels.

### 2.3 Diagnostic standards

The standards in the Chinese Adult Dyslipidemia Prevention Guide (2007 Edition) were used for diagnosis of lipid metabolism abnormalities. The ALOKA SSD-5000 color Doppler ultrasonography scanner was used to determine whether gallstones were present. A frequency of 3.5 MHz was used for the ultrasonography. Gallstones present as 1 or more visible acoustic shadows with irregular size in the gallbladder lumen; strong acoustic shadows may move depending on the position of the patient. All patients with gallstones underwent computed tomography for diagnostic confirmation.

### 2.4 Statistical analysis

All raw data were collected by using an Excel spreadsheet. SPSS 20.0 (Chicago, IL) was used for the data analysis. Continuous normally distributed data were presented as mean ± standard deviation, and the *t* test was used for comparisons between the groups. The Chi-square test was used for categorical variables. Factors that affect gallstone formation were first analyzed by using univariate logistic regression, and indexes with statistical significance were included in the multivariate logistic regression to confirm that they are independent factors affecting gallstone formation. All reported *P*-values are 2-sided, and *P*-values < .05 were considered statistically significant for all included studies.

## 3 Results

### 3.1 Clinical and demographic characteristics of the physical examinees

Among the 2288 participants examined, 1237 were men, and the remaining 1051 were women. The mean age of the subjects was 47.4 years in participants without gallstones, and 50.4 years in subjects with gallstones. The total incidence of gallstones was 4.11% (94/2288). Overall, there were significant differences between the gallstone and nongallstone groups regarding the characteristics of mean age (*P* = .038), weight (*P* = .021), ALT (*P* = .048), total SB (*P* < .001), free SB (*P* < .001), TG (*P* < .001), and LDL (*P* = .001). Further, there were no significant differences for sex, educational level, marital status, waist circumference, TC, HDL, and FBG (Table 1).

**Table 1:**
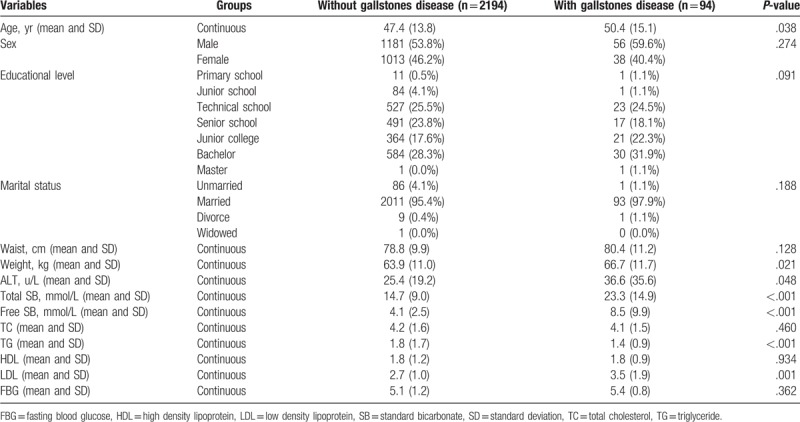
Baseline characteristics of recruited adults.

### 3.2 Indexes associated with gallstones

The results of the univariate logistic regression analysis are presented in Table 2. Overall, we noted that sex was not associated with the incidence of gallstone disease (*P* = .275), while age was significantly associated with the incidence of this condition (*P* = .039). However, educational level, marital status, waist circumference, TC, HDL, and FBG were not associated with the risk of developing gallstones. Among the physical examinations and blood markers, we noted that weight (odds ratio [OR]: 1.02; 95% confidence interval [CI]: 1.00–1.04; *P* = .021), ALT (OR: 1.02; 95% CI: 1.01–1.03; *P* = .001), total SB (OR: 1.04; 95% CI: 1.02–1.06; *P* < .001), free SB (OR: 1.17; 95% CI: 1.12–1.21; *P* < .001), TG (OR: 0.76; 95% CI: 0.60–0.97; *P* = .024), and LDL (OR: 1.59; 95% CI: 1.32–1.91; *P* < .001) correlated with the risk of developing gallstones.

**Table 2:**
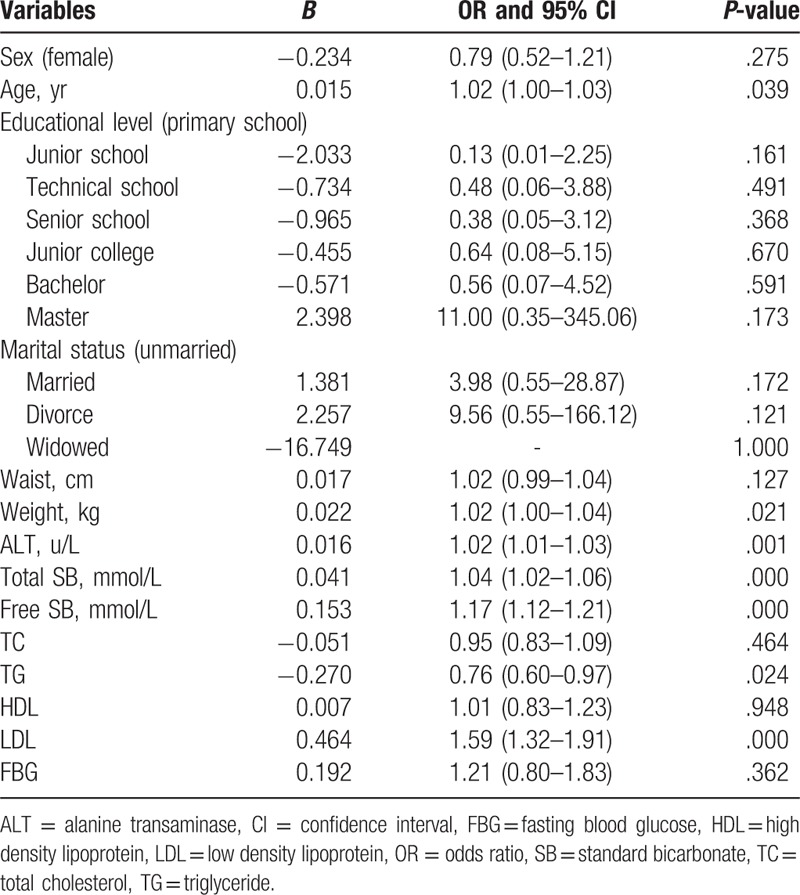
Unadjusted factors and the risk of gallstone disease.

Multivariate logistic regression analysis was used to confirm that the aforementioned factors independently affect the incidence of gallstone disease. We noted increased LDL levels were associated with an increased risk of gallstone disease (OR: 1.92; 95% CI: 1.31–2.81; *P* < .001). However, other factors including age, weight, ALT, total SB, free SB, and TG were not associated with an increased risk of gallstone disease (Table 3).

**Table 3:**
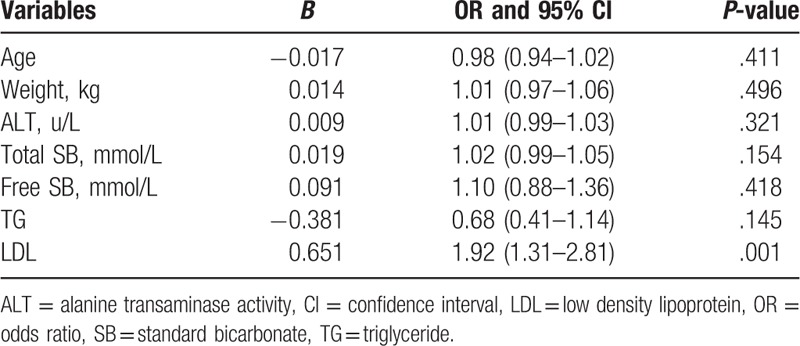
Continues adjusted factors and the risk of gallstone disease.

## 4 Discussion

Previous studies have already illustrated the role of females sex,^[12]^ obesity,^[13]^ elder age,^[14]^ metabolic syndrome,^[15,16]^ and viral infection^[17]^ in the risk of gallstone disease. However, the potential risk factors for the incidence of gallstone disease in China were not investigated. The crude results of this study found there were significant differences between the gallstone and nongallstone groups with respect to age, weight, ALT, total SB, free SB, TG, and LDL, although after sequential adjustment included other factors, increased LDL levels were associated with an increased risk of developing gallstones, and there were no significant associations for other factors.

The crude results of this study indicated age, weight, ALT, total SB, free SB, TG, and LDL were associated with the incidence of gallstone disease. The study conducted by Festi et al indicated that age, body mass index, diabetes, peptic ulcer and angina, TC, and TG were associated with the risk of gallstone development in men, while only age and body mass index were risk factors in women.^[14]^ The reason for this could be that those factors are characterizing metabolic syndrome, which correlates with the risk of gallstone disease.^[18,19]^ In this study, after continuous adjustments for patients’ characteristics, only LDL was associated with increased risk of gallstone disease. The potential reason for this could be the lower incidence of gallstone formation associated with a wide range of 95% CIs. Moreover, several important characteristics including dietary habits, history of gallstone disease in the participant's first-degree relatives, history of gastrointestinal surgery, parity and use of oral contraceptives, smoking, alcohol consumption, use of hypolipidemic drugs, and environmental influences were not available, which might affect the progression of gallstone disease.

Previous studies illustrated that abnormalities in lipid metabolism or liver function might affect the progression of gallstone disease, and abnormal blood markers correlated with lipid metabolism and liver function, which should be evaluated to determine the relationship between lipid profiles and gallstone formation.^[20]^ Therefore, the present study focused on individuals in the Jinshan district of Shanghai who underwent successive physical examinations, and TC, TG, LDL, HDL, and ALT were chosen as parameters for evaluating the incidence of gallstone disease to identify the blood markers correlated with its development. Results of the multivariate analysis found only the LDL levels were independent factors for the incidence of gallstone disease, and no other significant factors were observed. Previous studies indicated increased cholesterol levels could induce the expressions of inflammatory factors such as interleukin (IL)-1, IL-6, and tumor necrosis factor-alpha, which could cause cholecystitis and induce the formation of gallstones.^[12,21]^

Liver function markers reflect the health status of the liver, and patients with increased ALT levels may develop abnormal liver function. Previous studies have already illustrated that nonalcoholic fatty liver disease is associated with the progression of gallstone formation.^[22,23]^ Furthermore, hepatitis C virus infection and cirrhosis are associated with the development of gallstones.^[24]^ An increased serum ALT level upon hospital admission could predict the risk of gallstone disease due to transient ampullary obstruction causing a rapid increase in bile duct pressure and consequent liver cell damage. Therefore, patients with gallstone disease are more likely to exhibit abnormal liver function. The present study could not demonstrate the causal association between ALT levels and the risk of gallstone disease due to its retrospective design and limited number of patients (only 4.11% were diagnosed with gallstone disease).

Although the findings of this study were not based on a large sample size, the results were stable. Several limitations of this study should be highlighted:

(1) the types of gallstone classifications were not recorded due to difficulty with distinguishing the gallstone types;
(2) the prevalence of gallstone disease was lower, which could affect the power to explore the risk factors for the progression of gallstone formation; and
(3) retrospective studies cannot adequately control patient conditions, including background, lipid-reducing drugs used, and other characteristics.

In conclusion, the incidence of gallstone disease was 4.11% in Shanghai City. The crude data suggested older age, higher body weight, ALT, total SB, free SB, and LDL were significantly correlated with the greater incidence of gallstone development, whereas increased TG was associated with a reduced risk of gallstone disease. After adjusting for potential factors, increased LDL levels were associated with an increased risk of gallstone disease. Large prospective studies should be conducted to verify these results and further discover the factors involved in the mechanism of gallstone formation.

## Author contributions

**Conceptualization:** Qiyun Gu.

**Data curation:** Guoqing Zhou.

**Formal analysis:** Qiyun Gu.

**Funding acquisition:** Qiyun Gu.

**Investigation:** Guoqing Zhou, Tao Xu.

**Methodology:** Qiyun Gu, Tao Xu.

**Resources:** Guoqing Zhou.

**Software:** Tao Xu.

**Supervision:** Qiyun Gu.

**Validation:** Guoqing Zhou.

**Visualization:** Tao Xu.

**Writing – original draft:** Qiyun Gu.

**Writing – review and editing:** Qiyun Gu.
